# {*N*-[1-(2-Oxidophen­yl)ethyl­idene]-dl-alaninato}(pentane-1,5-di­yl)silicon(IV)

**DOI:** 10.1107/S2414314624002281

**Published:** 2024-03-19

**Authors:** Uwe Böhme, Sabine Fels

**Affiliations:** a Institut für Anorganische Chemie, Technische Universität Bergakademie Freiberg, Leipziger Str. 29, 09599 Freiberg, Germany; Sunway University, Malaysia

**Keywords:** crystal structure, silicon complex, Schiff base ligand, penta­coordination

## Abstract

The title Si^IV^ complex, C_16_H_21_NO_3_Si, is built up by a tridentate dinegative Schiff base ligand bound to a sila­cyclo­hexane unit. The coordination geometry of the penta­coordinated Si^IV^ atom is a distorted trigonal bipyramid. The presence of the sila­cyclo­hexane ring in the complex leads to an unusual coordination geometry of the Si^IV^ atom with the N atom from the Schiff base ligand and an alkyl-C atom in apical positions of the trigonal bipyramid. There is a disorder of the methyl group at the imine bond with two orientations resolved for the H atoms [major orientation = 0.55 (3)]. In the crystal, C—H⋯O inter­actions are found within corrugated layers of mol­ecules parallel to the *ab* plane.

## Abstract 2

The coordination geometry of the penta­coordinated Si^IV^ atom in the title complex is a distorted trigonal bipyramid.

## Graphical abstract

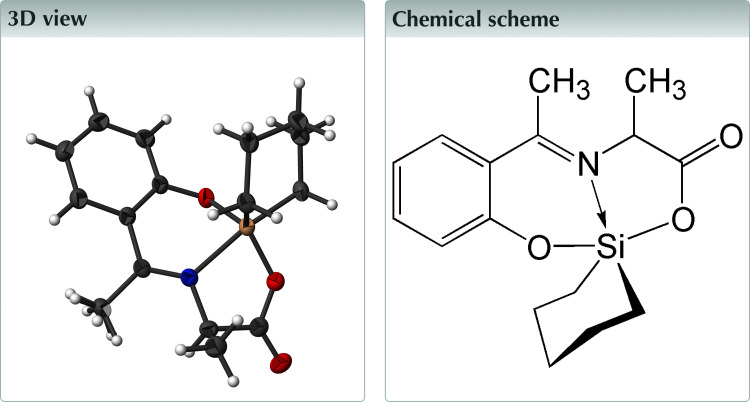

## Structure description

Schiff base ligands with additional O donor ligands are suitable ligands to generate penta­coordinated Si^IV^ complexes (Wagler *et al.*, 2014 ▸). The Schiff base *N*-[1-(2-hy­droxy­phen­yl)ethyl­idene]-dl-alanine has been utilized previously for the preparation of a Cu^II^ complex (Zhao *et al.*, 2008 ▸). The Cu^II^ atom of this complex is coordinated to the tridentate Schiff base ligand and the bidentate bis­(3,5-di­methyl­pyrazol-1-yl)methane ligand. This Schiff base ligand has not yet been used for the generation of silicon complexes. Schiff base ligands derived from salicyl aldehyde (Warncke *et al.*, 2012 ▸), *o*-hy­droxy­aceto­phenone (Böhme *et al.*, 2006 ▸) and naphthyl aldehyde (Schwarzer *et al.*, 2018 ▸) have been used for the preparation of related Si^IV^ complexes.

**Figure 1:**
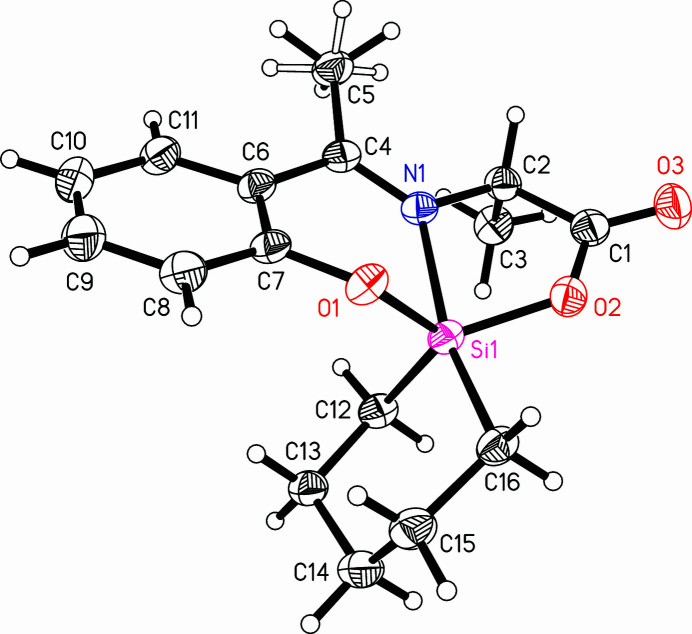
A view of the mol­ecular structure of the title compound, with the atom-labeling scheme. Displacement ellipsoids are drawn at the 50% probability level.

The title compound, C_16_H_21_NO_3_Si, crystallizes with one molecule in the asymmetric unit (Fig. 1 ▸). The Schiff base ligand is formally dinegatively charged and coordinates the Si^IV^ atom *via* the phen­oxy-O1, imine-N1 and carboxyl-O2 atoms. The Si^IV^ atom is part of a sila­cyclo­hexane ring and is bound therein *via* the C12 and C16 atoms. The coordination geometry of this penta­coordinate Si^IV^ complex was analyzed with the parameter τ (Addison *et al.*, 1984 ▸). The largest bond angle *β* and the second largest angle *α* at the Si^IV^ atom are used to calculate this parameter with *τ* = (*β* − *α*)/60°. A value of τ = 0 indicates a perfect square pyramid, whereas a value of τ = 1 indicates a perfect trigonal bipyramid. In the complex under investigation the largest angle at the Si^IV^ atom is C16—Si1—N1 with 167.09 (8)°. The second largest angle is O1—Si1—O2 with 123.53 (7)° (see Table 1 ▸). This leads to a parameter τ = 0.73, which corresponds to a distorted trigonal bipyramid. The apical positions are represented by N1 and C16, while the atoms O1, O2 and C12 are the atoms in the trigonal plane. Silicon complexes with tridentate O,*N*,*O*′-Schiff base ligands and two alkyl groups form mainly distorted trigonal bipyramidal geometries in the solid state (Schwarzer *et al.*, 2018 ▸; Böhme & Fels, 2023*a* ▸,*b* ▸). The apical positions of the coordination polyhedron are usually occupied by the two O atoms of the Schiff base, when there are two single alkyl groups bound to the Si^IV^ atom. Having N and C atoms in apical positions has so far only been observed in the case of a sila­cyclo­butane derivative (Schwarzer *et al.*, 2018 ▸). The sila­cyclo­hexane ring in the complex under investigation leads to a similar coordination geometry as in the sila­cyclo­butane derivative.

The Cremer–Pople puckering parameters (Cremer & Pople, 1975 ▸) for the six-membered sila­cyclo­hexane ring are *Q* = 0.619 (2) Å, Θ = 177.96 (19)° and φ = 244 (3)°, which is indicative of a chair conformation (Boeyens, 1978 ▸).

The Si1–O1 bond [1.7029 (13) Å] is shorter than Si1–O2 [1.7474 (14) Å], which is easily explained by the electronegative character of the phenyl bound O1 atom and the carboxyl type O2 atom. The Si1—C and Si1—N1 bonds, Table 1 ▸, have similar lengths to those in comparable penta­coordinate silicon complexes (Böhme *et al.*, 2006 ▸; Böhme & Günther, 2007 ▸; Böhme & Föhn, 2007 ▸; Schwarzer *et al.*, 2018 ▸).

Inter­molecular inter­actions are observed between C2—H2⋯O3 and C12—H12*B*⋯O3, Table 2 ▸. The hydrogen bonds lead to corrugated layers of mol­ecules lying parallel to the crystallographic *ab* plane.

## Synthesis and crystallization

The sodium salt of the Schiff base ligand was prepared from 2-hy­droxy­aceto­phenone and alanine according to a literature procedure (Fels, 2015 ▸). To a solution of 1.12 g (4.89 mmol) sodium{*N*-[1-(2-hy­droxy­phen­yl)ethyl­idene]-dl-alaninate} in 30 ml of dry THF was added 0.64 g (6.36 mmol) tri­ethyl­amine, which led to a yellow suspension. The ClSiMe_3_ (1.27 g, 11.74 mmol) precursor was added with a syringe *via* a septum. A white precipitate of tri­ethyl­ammonium chloride formed during stirring at 50°C for 1 h. The tri­ethyl­ammonium chloride was filtered off and the residue was washed with 10 ml THF. The filtrate was reduced in a vacuum and was carefully freed from volatile components at 90°C in a vacuum. The remaining pale-yellow liquid was dissolved in 20 ml of THF. 1,1-Di­chloro­sila­cyclo­hexane (0.71 g, 4.18 mmol) was diluted with 5 ml of THF and added with a syringe to the solution. A pale-yellow suspension formed. This suspension was stirred for 20 days at room temperature. Filtration of the suspension gave a pale-yellow solution, which was reduced in a vacuum to a gray–brown solid. Recrystallization from the mixed solvents of chloro­form (8 ml) and *n*-hexane (5 ml) yielded pale-yellow crystals suitable for crystal structure analysis, yield: 0.57 g (45%), m.p. = 435 K.

^1^H NMR (400 MHz, CDCl_3_) δ (p.p.m.): 1.49 (*m*, 3H, CH-**CH_3_**), 0.49–1.87 (*mm*, 10H, CH_2_), 2.47 (*s*, 3H, **CH_3_**—C=N), 4.30 (*m*, 1H, **CH**—COO), 6.94 (*m*, 1H, H_ar_); 7.00 (*m*, 1H, H_ar_); 7.41 (*m*, 1H, H_ar_); 7.51 (*m*, 1H, H_ar_); ^13^C NMR (101 MHz, CDCl_3_) δ (p.p.m.): 17.2 (**C**H_3_—C=N), 17.9 (**C**H_2_—Si—**C**H_2_), 19.5 (CH—**C**H_3_), 24.8, 25.1, 28.8 (3 CH_2_), 56.6 (**C**H—COO), 119.5, 120.0, 121.1, 127.6, 134.5 (5 C_ar_), 157.9 (CH=N), 170.5 (C_ar_—O), 171.3 (COO); ^29^Si NMR (CDCl_3_, 79.5 MHz) δ (p.p.m.): −67.0.

## Refinement

Crystal data, data collection and structure refinement details for the title compound are summarized in Table 3 ▸. There is disorder at the C5-methyl group, which was resolved with two positions of the methyl-H atoms; the major orientation had a site occupancy of 0.55 (3).

## Supplementary Material

Crystal structure: contains datablock(s) I. DOI: 10.1107/S2414314624002281/tk4102sup1.cif

Structure factors: contains datablock(s) I. DOI: 10.1107/S2414314624002281/tk4102Isup2.hkl

CCDC reference: 2338755

Additional supporting information:  crystallographic information; 3D view; checkCIF report

## Figures and Tables

**Table 1:** Selected geometric parameters (Å, °)

Si1—O1	1.7029 (13)	Si1—C16	1.8961 (19)
Si1—O2	1.7474 (14)	Si1—N1	2.0883 (16)
Si1—C12	1.8804 (19)		
			
O1—Si1—O2	123.53 (7)	C12—Si1—C16	99.85 (8)
O1—Si1—C12	112.99 (8)	O1—Si1—N1	85.08 (6)
O2—Si1—C12	121.46 (8)	O2—Si1—N1	79.30 (6)
O1—Si1—C16	94.47 (8)	C12—Si1—N1	92.16 (7)
O2—Si1—C16	90.31 (8)	C16—Si1—N1	167.09 (8)

**Table 2:** Hydrogen-bond geometry (Å, °)

*D*—H⋯*A*	*D*—H	H⋯*A*	*D*⋯*A*	*D*—H⋯*A*
C2—H2⋯O3^i^	0.97 (2)	2.45 (2)	3.197 (2)	133.1 (17)
C12—H12*B*⋯O3^ii^	0.99	2.64	3.571 (2)	155

Symmetry codes: (i) 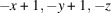 ; (ii) 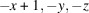 .

**Table 3:** Experimental details

Crystal data	
Chemical formula	C_16_H_21_NO_3_Si
*M*_r_	303.43
Crystal system, space group	Triclinic, *P* 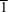
Temperature (K)	173
*a*, *b*, *c* (Å)	6.7236 (5), 7.2935 (5), 16.1649 (11)
α, β, γ (°)	77.570 (6), 80.354 (5), 89.412 (6)
*V* (Å^3^)	762.90 (10)
*Z*	2
Radiation type	Mo *K*α
μ (mm^−1^)	0.16
Crystal size (mm)	0.30 × 0.15 × 0.05

Data collection	
Diffractometer	STOE *IPDS* 2
Absorption correction	Integration (*X-RED*; Stoe & Cie, 2009 ▸)
*T*_min_, *T*_max_	0.832, 0.984
No. of measured, independent and observed [*I* > 2σ(*I*)] reflections	21687, 3289, 2700
*R*_int_	0.090
(sin θ/λ)_max_ (Å^−1^)	0.637

Refinement	
*R*[*F* ^2^ > 2σ(*F* ^2^)], *wR*(*F* ^2^), *S*	0.042, 0.107, 1.10
No. of reflections	3289
No. of parameters	197
H-atom treatment	H atoms treated by a mixture of independent and constrained refinement
Δρ_max_, Δρ_min_ (e Å^−3^)	0.37, −0.28

Computer programs: *X-AREA* and *X-RED* (Stoe & Cie, 2009 ▸), *SHELXS97* and *SHELXL97* (Sheldrick, 2008 ▸), and *ORTEP-3 for Windows* (Farrugia, 2012 ▸).

## full crystallographic data

### Crystal data

**Table:**

C_16_H_21_NO_3_Si	*Z* = 2
*M**_r_* = 303.43	*F*(000) = 324
Triclinic, *P*1	*D*_x_ = 1.321 Mg m^−^^3^
*a* = 6.7236 (5) Å	Mo *K*α radiation, λ = 0.71073 Å
*b* = 7.2935 (5) Å	Cell parameters from 21687 reflections
*c* = 16.1649 (11) Å	θ = 2.6–27.3°
α = 77.570 (6)°	µ = 0.16 mm^−^^1^
β = 80.354 (5)°	*T* = 173 K
γ = 89.412 (6)°	Prism, colourless
*V* = 762.90 (10) Å^3^	0.30 × 0.15 × 0.05 mm

### Data collection

**Table:**

STOE IPDS 2 diffractometer	3289 independent reflections
Radiation source: fine-focus sealed tube	2700 reflections with *I* > 2σ(*I*)
Graphite monochromator	*R*_int_ = 0.090
Detector resolution: 6.67 pixels mm^-1^	θ_max_ = 26.9°, θ_min_ = 2.6°
rotation method, ω scans	*h* = −8→8
Absorption correction: integration (X-RED; Stoe & Cie, 2009)	*k* = −9→9
*T*_min_ = 0.832, *T*_max_ = 0.984	*l* = −20→20
21687 measured reflections	

### Refinement

**Table:**

Refinement on *F*^2^	Primary atom site location: structure-invariant direct methods
Least-squares matrix: full	Hydrogen site location: mixed
*R*[*F*^2^ > 2σ(*F*^2^)] = 0.042	H atoms treated by a mixture of independent and constrained refinement
*wR*(*F*^2^) = 0.107	*w* = 1/[σ^2^(*F*_o_^2^) + (0.0375*P*)^2^ + 0.4037*P*] where *P* = (*F*_o_^2^ + 2*F*_c_^2^)/3
*S* = 1.10	(Δ/σ)_max_ < 0.001
3289 reflections	Δρ_max_ = 0.37 e Å^−^^3^
197 parameters	Δρ_min_ = −0.28 e Å^−^^3^
0 restraints	

### Special details

**Table:**

Geometry. All esds (except the esd in the dihedral angle between two l.s. planes) are estimated using the full covariance matrix. The cell esds are taken into account individually in the estimation of esds in distances, angles and torsion angles; correlations between esds in cell parameters are only used when they are defined by crystal symmetry. An approximate (isotropic) treatment of cell esds is used for estimating esds involving l.s. planes.

### Fractional atomic coordinates and isotropic or equivalent isotropic displacement parameters (Å^2^)

**Table:**

	*x*	*y*	*z*	*U*_iso_*/*U*_eq_	Occ. (<1)
Si1	0.46505 (7)	0.07538 (7)	0.20401 (3)	0.02309 (14)	
O1	0.44797 (19)	0.20645 (18)	0.28018 (9)	0.0275 (3)	
O2	0.5843 (2)	0.15539 (19)	0.09769 (8)	0.0298 (3)	
O3	0.5822 (2)	0.2919 (2)	−0.04004 (9)	0.0378 (3)	
N1	0.2467 (2)	0.2525 (2)	0.15507 (10)	0.0238 (3)	
C1	0.4944 (3)	0.2453 (3)	0.03353 (12)	0.0284 (4)	
C2	0.2731 (3)	0.2819 (3)	0.06087 (12)	0.0263 (4)	
H2	0.255 (3)	0.412 (3)	0.0344 (14)	0.031 (6)*	
C3	0.1425 (3)	0.1443 (3)	0.03230 (13)	0.0325 (4)	
H3A	0.167965	0.015503	0.060743	0.049*	
H3B	0.176307	0.158255	−0.030152	0.049*	
H3C	−0.000290	0.171095	0.048014	0.049*	
C4	0.1068 (3)	0.3338 (2)	0.19815 (12)	0.0249 (4)	
C5	−0.0402 (3)	0.4663 (3)	0.15733 (13)	0.0334 (4)	
H5A	0.032877	0.556000	0.107753	0.050*	0.55 (3)
H5B	−0.109387	0.534539	0.199364	0.050*	0.55 (3)
H5C	−0.139702	0.394647	0.138328	0.050*	0.55 (3)
H5D	−0.027028	0.463040	0.096368	0.050*	0.45 (3)
H5E	−0.011114	0.594190	0.162767	0.050*	0.45 (3)
H5F	−0.178071	0.427956	0.186311	0.050*	0.45 (3)
C6	0.0976 (3)	0.2987 (2)	0.29215 (12)	0.0252 (4)	
C7	0.2700 (3)	0.2417 (2)	0.32842 (12)	0.0254 (4)	
C8	0.2650 (3)	0.2224 (3)	0.41654 (13)	0.0321 (4)	
H8	0.383339	0.188702	0.440518	0.039*	
C9	0.0885 (3)	0.2519 (3)	0.46913 (14)	0.0375 (5)	
H9	0.086339	0.237791	0.529089	0.045*	
C10	−0.0865 (3)	0.3024 (3)	0.43487 (14)	0.0368 (5)	
H10	−0.208071	0.320055	0.471404	0.044*	
C11	−0.0815 (3)	0.3263 (3)	0.34729 (13)	0.0310 (4)	
H11	−0.200135	0.361978	0.323846	0.037*	
C12	0.2849 (3)	−0.1331 (2)	0.23573 (12)	0.0270 (4)	
H12A	0.145209	−0.089668	0.234512	0.032*	
H12B	0.316084	−0.214906	0.193935	0.032*	
C13	0.3009 (3)	−0.2467 (3)	0.32707 (13)	0.0293 (4)	
H13A	0.264948	−0.164827	0.368583	0.035*	
H13B	0.201379	−0.352790	0.342705	0.035*	
C14	0.5115 (3)	−0.3243 (3)	0.33537 (14)	0.0349 (5)	
H14A	0.505593	−0.406052	0.393243	0.042*	
H14B	0.548839	−0.403187	0.292686	0.042*	
C15	0.6765 (3)	−0.1726 (3)	0.32199 (14)	0.0333 (4)	
H15A	0.805711	−0.233738	0.330840	0.040*	
H15B	0.641747	−0.095644	0.365645	0.040*	
C16	0.7044 (3)	−0.0436 (3)	0.23210 (13)	0.0284 (4)	
H16A	0.754746	−0.118478	0.188979	0.034*	
H16B	0.808546	0.054194	0.228408	0.034*	

### Atomic displacement parameters (Å^2^)

**Table:**

	*U*^11^	*U*^22^	*U*^33^	*U*^12^	*U*^13^	*U*^23^
Si1	0.0225 (2)	0.0213 (2)	0.0264 (3)	0.00193 (18)	−0.00397 (19)	−0.00730 (18)
O1	0.0249 (6)	0.0293 (7)	0.0323 (7)	0.0046 (5)	−0.0078 (5)	−0.0133 (5)
O2	0.0266 (7)	0.0327 (7)	0.0284 (7)	0.0029 (5)	−0.0019 (5)	−0.0051 (5)
O3	0.0418 (8)	0.0396 (8)	0.0284 (7)	−0.0018 (6)	0.0026 (6)	−0.0062 (6)
N1	0.0259 (8)	0.0206 (7)	0.0258 (8)	0.0009 (6)	−0.0055 (6)	−0.0061 (6)
C1	0.0330 (10)	0.0235 (8)	0.0294 (10)	−0.0014 (7)	−0.0039 (8)	−0.0085 (7)
C2	0.0316 (10)	0.0244 (9)	0.0235 (9)	0.0017 (7)	−0.0060 (7)	−0.0054 (7)
C3	0.0346 (10)	0.0344 (10)	0.0314 (10)	0.0003 (8)	−0.0086 (8)	−0.0110 (8)
C4	0.0259 (9)	0.0203 (8)	0.0293 (9)	0.0013 (7)	−0.0063 (7)	−0.0062 (7)
C5	0.0352 (11)	0.0316 (10)	0.0339 (10)	0.0115 (8)	−0.0088 (9)	−0.0064 (8)
C6	0.0288 (9)	0.0216 (8)	0.0272 (9)	0.0027 (7)	−0.0056 (7)	−0.0091 (7)
C7	0.0294 (9)	0.0189 (8)	0.0294 (9)	0.0029 (7)	−0.0056 (7)	−0.0082 (7)
C8	0.0372 (11)	0.0312 (10)	0.0316 (10)	0.0052 (8)	−0.0122 (8)	−0.0101 (8)
C9	0.0478 (13)	0.0388 (11)	0.0280 (10)	0.0071 (9)	−0.0080 (9)	−0.0108 (8)
C10	0.0385 (11)	0.0400 (11)	0.0309 (10)	0.0073 (9)	0.0002 (9)	−0.0105 (9)
C11	0.0309 (10)	0.0305 (9)	0.0329 (10)	0.0066 (8)	−0.0060 (8)	−0.0099 (8)
C12	0.0263 (9)	0.0238 (8)	0.0326 (10)	0.0022 (7)	−0.0069 (7)	−0.0082 (7)
C13	0.0294 (9)	0.0224 (8)	0.0341 (10)	0.0021 (7)	−0.0021 (8)	−0.0045 (7)
C14	0.0350 (11)	0.0286 (10)	0.0391 (11)	0.0064 (8)	−0.0074 (9)	−0.0027 (8)
C15	0.0304 (10)	0.0344 (10)	0.0365 (11)	0.0072 (8)	−0.0101 (8)	−0.0072 (8)
C16	0.0224 (9)	0.0275 (9)	0.0360 (10)	0.0037 (7)	−0.0052 (8)	−0.0086 (8)

### Geometric parameters (Å, º)

**Table:**

Si1—O1	1.7029 (13)	C6—C7	1.404 (3)
Si1—O2	1.7474 (14)	C6—C11	1.413 (3)
Si1—C12	1.8804 (19)	C7—C8	1.396 (3)
Si1—C16	1.8961 (19)	C8—C9	1.383 (3)
Si1—N1	2.0883 (16)	C8—H8	0.9500
O1—C7	1.369 (2)	C9—C10	1.396 (3)
O2—C1	1.334 (2)	C9—H9	0.9500
O3—C1	1.214 (2)	C10—C11	1.383 (3)
N1—C4	1.291 (2)	C10—H10	0.9500
N1—C2	1.472 (2)	C11—H11	0.9500
C1—C2	1.518 (3)	C12—C13	1.550 (3)
C2—C3	1.532 (3)	C12—H12A	0.9900
C2—H2	0.97 (2)	C12—H12B	0.9900
C3—H3A	0.9800	C13—C14	1.536 (3)
C3—H3B	0.9800	C13—H13A	0.9900
C3—H3C	0.9800	C13—H13B	0.9900
C4—C6	1.476 (3)	C14—C15	1.531 (3)
C4—C5	1.507 (3)	C14—H14A	0.9900
C5—H5A	0.9800	C14—H14B	0.9900
C5—H5B	0.9800	C15—C16	1.535 (3)
C5—H5C	0.9800	C15—H15A	0.9900
C5—H5D	0.9800	C15—H15B	0.9900
C5—H5E	0.9800	C16—H16A	0.9900
C5—H5F	0.9800	C16—H16B	0.9900
			
O1—Si1—O2	123.53 (7)	C11—C6—C4	121.21 (17)
O1—Si1—C12	112.99 (8)	O1—C7—C8	117.48 (17)
O2—Si1—C12	121.46 (8)	O1—C7—C6	122.46 (17)
O1—Si1—C16	94.47 (8)	C8—C7—C6	120.05 (17)
O2—Si1—C16	90.31 (8)	C9—C8—C7	120.32 (19)
C12—Si1—C16	99.85 (8)	C9—C8—H8	119.8
O1—Si1—N1	85.08 (6)	C7—C8—H8	119.8
O2—Si1—N1	79.30 (6)	C8—C9—C10	120.56 (19)
C12—Si1—N1	92.16 (7)	C8—C9—H9	119.7
C16—Si1—N1	167.09 (8)	C10—C9—H9	119.7
C7—O1—Si1	123.83 (12)	C11—C10—C9	119.46 (19)
C1—O2—Si1	125.22 (12)	C11—C10—H10	120.3
C4—N1—C2	121.84 (16)	C9—C10—H10	120.3
C4—N1—Si1	126.93 (13)	C10—C11—C6	120.99 (19)
C2—N1—Si1	111.20 (11)	C10—C11—H11	119.5
O3—C1—O2	122.73 (18)	C6—C11—H11	119.5
O3—C1—C2	123.35 (18)	C13—C12—Si1	110.69 (13)
O2—C1—C2	113.92 (16)	C13—C12—H12A	109.5
N1—C2—C1	104.45 (15)	Si1—C12—H12A	109.5
N1—C2—C3	112.04 (15)	C13—C12—H12B	109.5
C1—C2—C3	109.48 (15)	Si1—C12—H12B	109.5
N1—C2—H2	111.4 (13)	H12A—C12—H12B	108.1
C1—C2—H2	105.5 (13)	C14—C13—C12	113.67 (16)
C3—C2—H2	113.3 (13)	C14—C13—H13A	108.8
C2—C3—H3A	109.5	C12—C13—H13A	108.8
C2—C3—H3B	109.5	C14—C13—H13B	108.8
H3A—C3—H3B	109.5	C12—C13—H13B	108.8
C2—C3—H3C	109.5	H13A—C13—H13B	107.7
H3A—C3—H3C	109.5	C15—C14—C13	114.03 (16)
H3B—C3—H3C	109.5	C15—C14—H14A	108.7
N1—C4—C6	117.34 (16)	C13—C14—H14A	108.7
N1—C4—C5	123.61 (17)	C15—C14—H14B	108.7
C6—C4—C5	119.02 (16)	C13—C14—H14B	108.7
C4—C5—H5A	109.5	H14A—C14—H14B	107.6
C4—C5—H5B	109.5	C14—C15—C16	112.77 (17)
H5A—C5—H5B	109.5	C14—C15—H15A	109.0
C4—C5—H5C	109.5	C16—C15—H15A	109.0
H5A—C5—H5C	109.5	C14—C15—H15B	109.0
H5B—C5—H5C	109.5	C16—C15—H15B	109.0
C4—C5—H5D	109.5	H15A—C15—H15B	107.8
C4—C5—H5E	109.5	C15—C16—Si1	113.81 (13)
H5D—C5—H5E	109.5	C15—C16—H16A	108.8
C4—C5—H5F	109.5	Si1—C16—H16A	108.8
H5D—C5—H5F	109.5	C15—C16—H16B	108.8
H5E—C5—H5F	109.5	Si1—C16—H16B	108.8
C7—C6—C11	118.54 (17)	H16A—C16—H16B	107.7
C7—C6—C4	120.22 (17)		
			
O2—Si1—O1—C7	125.72 (14)	Si1—O1—C7—C8	134.77 (15)
C12—Si1—O1—C7	−38.25 (16)	Si1—O1—C7—C6	−46.4 (2)
C16—Si1—O1—C7	−140.93 (14)	C11—C6—C7—O1	178.09 (16)
N1—Si1—O1—C7	52.01 (14)	C4—C6—C7—O1	−3.7 (3)
O1—Si1—O2—C1	−90.30 (16)	C11—C6—C7—C8	−3.1 (3)
C12—Si1—O2—C1	72.36 (16)	C4—C6—C7—C8	175.16 (16)
C16—Si1—O2—C1	174.13 (15)	O1—C7—C8—C9	−178.58 (17)
N1—Si1—O2—C1	−13.60 (14)	C6—C7—C8—C9	2.5 (3)
Si1—O2—C1—O3	−177.12 (14)	C7—C8—C9—C10	−0.3 (3)
Si1—O2—C1—C2	2.3 (2)	C8—C9—C10—C11	−1.3 (3)
C4—N1—C2—C1	154.00 (16)	C9—C10—C11—C6	0.7 (3)
Si1—N1—C2—C1	−24.26 (16)	C7—C6—C11—C10	1.5 (3)
C4—N1—C2—C3	−87.6 (2)	C4—C6—C11—C10	−176.72 (18)
Si1—N1—C2—C3	94.15 (16)	O1—Si1—C12—C13	−50.11 (14)
O3—C1—C2—N1	−164.67 (17)	O2—Si1—C12—C13	145.55 (12)
O2—C1—C2—N1	15.9 (2)	C16—Si1—C12—C13	49.07 (14)
O3—C1—C2—C3	75.2 (2)	N1—Si1—C12—C13	−135.68 (13)
O2—C1—C2—C3	−104.25 (18)	Si1—C12—C13—C14	−60.40 (18)
C2—N1—C4—C6	−178.26 (15)	C12—C13—C14—C15	64.6 (2)
Si1—N1—C4—C6	−0.3 (2)	C13—C14—C15—C16	−61.2 (2)
C2—N1—C4—C5	−0.6 (3)	C14—C15—C16—Si1	56.1 (2)
Si1—N1—C4—C5	177.39 (14)	O1—Si1—C16—C15	65.85 (15)
N1—C4—C6—C7	23.1 (2)	O2—Si1—C16—C15	−170.47 (14)
C5—C4—C6—C7	−154.68 (17)	C12—Si1—C16—C15	−48.42 (16)
N1—C4—C6—C11	−158.72 (17)	N1—Si1—C16—C15	153.3 (3)
C5—C4—C6—C11	23.5 (3)		

### Hydrogen-bond geometry (Å, º)

**Table:**

*D*—H···*A*	*D*—H	H···*A*	*D*···*A*	*D*—H···*A*
C2—H2···O3^i^	0.97 (2)	2.45 (2)	3.197 (2)	133.1 (17)
C12—H12*B*···O3^ii^	0.99	2.64	3.571 (2)	155

Symmetry codes: (i) −*x*+1, −*y*+1, −*z*; (ii) −*x*+1, −*y*, −*z*.
